# Nutrition support practices in critically ill head-injured patients: a global perspective

**DOI:** 10.1186/s13054-015-1177-1

**Published:** 2016-01-07

**Authors:** Lee-anne S. Chapple, Marianne J. Chapman, Kylie Lange, Adam M. Deane, Daren K. Heyland

**Affiliations:** 1 Discipline of Acute Care Medicine, University of Adelaide, North Terrace, Adelaide, South Australia 5000 Australia; 2 Intensive Care Unit, Level 4, Emergency Services Building, Royal Adelaide Hospital, North Terrace, Adelaide, South Australia 5000 Australia; 3 Discipline of Medicine, University of Adelaide, North Terrace, Adelaide, South Australia 5000 Australia; 4 Clinical Evaluation Research Unit, Kingston General Hospital, Kingston, Ontario Canada; 5 Queen’s University, Kingston, Ontario Canada

**Keywords:** Nutrition support, Nutritional status, Head injury, Head trauma, Traumatic brain injury, Critical illness

## Abstract

**Background:**

Critical illness following head injury is associated with a hypermetabolic state but there are insufficient epidemiological data describing acute nutrition delivery to this group of patients. Furthermore, there is little information describing relationships between nutrition and clinical outcomes in this population.

**Methods:**

We undertook an analysis of observational data, collected prospectively as part of International Nutrition Surveys 2007-2013, and extracted data obtained from critically ill patients with head trauma. Our objective was to describe global nutrition support practices in the first 12 days of hospital admission after head trauma, and to explore relationships between energy and protein intake and clinical outcomes. Data are presented as mean (SD), median (IQR), or percentages.

**Results:**

Data for 1045 patients from 341 ICUs were analyzed. The age of patients was 44.5 (19.7) years, 78 % were male, and median ICU length of stay was 13.1 (IQR 7.9-21.6) days. Most patients (94 %) were enterally fed but received only 58 % of estimated energy and 53 % of estimated protein requirements. Patients from an ICU with a feeding protocol had greater energy and protein intakes (p <0.001, 0.002 respectively) and were more likely to survive (OR 0.65; 95 % CI 0.42-0.99; p = 0.043) than those without. Energy or protein intakes were not associated with mortality. However, a greater energy and protein deficit was associated with longer times until discharge alive from both ICU and hospital (all p <0.001).

**Conclusion:**

Nutritional deficits are commonplace in critically ill head-injured patients and these deficits are associated with a delay to discharge alive.

## Background

Head-injured patients frequently have increases in metabolic rate and protein catabolism that could lead to elevated nutritional needs [1, 2]. Energy expenditure may increase to 200 % of usual values but factors such as delayed gastric emptying, interruptions to feeding due to fasting for medical interventions, and inadvertent removal of feeding tubes hinder the provision of adequate nutrition in these patients [1, 3–6]. This is associated with up to 30 % loss of body weight and signs of malnutrition in about two thirds of patients two months after hospital admission [7]. It is plausible that such physical signs are associated with clinical outcomes such as length of hospitalization [8].

Despite this, there is a paucity of epidemiological data that describe actual nutrition practices for critically ill patients post head injury and previous studies have generally included cohorts of relatively small numbers, with the majority of observations being retrospective and/or from single centres. Additionally, while patients may have poor nutritional status after head injury, the associations between nutrition delivery and clinical outcomes have rarely been explored.

Hitherto, the literature on nutrition after head injury focuses on the mode or timing of nutrient delivery, rather than energy or protein delivery [9, 10]. Only one study has evaluated relationships between nutrient intake and clinical outcomes: Hartl and colleagues reported that after severe head injury there appeared to be an early survival benefit associated with maximum daily energy intake [11]. However, the relationships between mean energy or protein intake and overall survival or morbidity outcomes, such as length of stay, have not been explored. Hence, it is understandable that guidelines on nutritional management of head-injured patients conclude that there are insufficient data to support specific recommendations on macronutrient intake [12–14]. Therefore, without evidence to support clinician decision-making it is likely that practice in the feeding of head-injured patients will vary greatly between institutions and countries.

A greater understanding of current feeding practices, including the use of feeding protocols, and the influence of nutrition support on recovery would be of benefit, particularly as head-injured patients are likely to stay in hospital for significant periods of time, allowing nutrition to influence outcomes. Therefore, we aimed to: (1) describe global nutrition practices after head injury in the first 12 days of ICU admission; (2) evaluate factors that influence nutrition delivery; and (3) explore the relationships between energy and protein intake and clinical outcomes in this cohort.

## Methods

We undertook a post-hoc subgroup analysis of observational data collected prospectively as part of the International Nutrition Survey (INS) from 592 participating ICUs conducted in the study years 2007, 2008, 2009, 2010, 2011, and 2013 (no survey took place in 2012). From this combined dataset we extracted data from all patients with a primary diagnosis of head trauma. Critically ill adult (≥18 years of age) patients who were mechanically ventilated within the first 48 hours of admission to the ICU and who remained in ICU for more than 72 hours were eligible. The full methodological details of the INS have been previously reported [15]. In brief, data were collected for the following variables: patient demographics; primary admission diagnosis; nutrition practices including energy and protein provision, estimated nutritional requirements, reasons for interruptions to feeding, and use of feeding protocols; dietetic involvement; and clinical outcomes including mortality, length of mechanical ventilation, and ICU/hospital length of stay. Nutrition data were collected from ICU admission for 12 days or until ICU discharge, with mortality assessed at hospital discharge or censored at day 60. Ethics approval for the INS was obtained from the Research Ethics Committee of the Queens University, Kingston, Ontario, in addition to local ethical approval from each participating site. Informed consent for data collected as part of the INS was waived.

Updates to the survey design that occurred over the six study years restricted the total population for some data variables. The presence of a baseline nutrition assessment and time to initiation of enteral nutrition from admission were collected from 2010 onwards only and the number of interruptions to enteral nutrition and lowest and highest daily blood glucose levels were recorded from 2009 onwards. Glasgow Coma Scale (GCS) and Sequential Organ Failure Assessment (SOFA) scores were first collected in 2013.

For the purposes of data collection hypoglycemic events were defined as a blood glucose concentration <3.5 mmol/l. Energy and protein intake data included that provided through enteral and parenteral routes on a daily basis. Data on administration of lipid as a proportion of propofol were also collected. However, data on nutrient from dextrose or oral intake were not collected.

### Statistical analysis

Statistical analyses were conducted using SPSS (v.22, IBM Inc). Categorical data are presented as counts and percentages, and continuous data are reported as mean (standard deviation) or median (range or interquartile range (IQR)) as appropriate. Energy and protein deficit was calculated as the mean daily absolute difference between intake and prescribed requirements. No adjustment was conducted for the amount of nutrition received on admission or discharge days shorter than 24 hours as in previous analyses of INS data [15]. Energy and protein intake, deficit, and the percentage of prescribed nutritional requirements that was met were calculated from all sources over all days before permanent progression to exclusive oral intake.

Pearson correlation was used to assess linear relationships between continuous variables. Associations with mortality and nutrition intake were determined by logistic regression and linear mixed effects models, respectively, adjusted for age, sex, region, Acute Physiology and Chronic Health Evaluation (APACHE II) score, body mass index (BMI) category, admission category, and clustering of patients within ICUs. Regions were categorized into Canada, USA, Australia and New Zealand, Europe and South Africa, Latin America and Asia. Admission category was defined as medical or surgical. Associations with mortality and nutrition intake were also adjusted for evaluable nutrition days. Evaluable nutrition days are defined as any day on which artificial nutrition was received or should have been provided, and excludes days when patients are transitioning to oral intake [16]. Time until discharge alive from ICU/hospital and length of mechanical ventilation were analysed using Cox proportional hazards regression, adjusted for age, sex, region, APACHE II score, BMI category, admission category, and clustering of patients within ICUs, with death in ICU/hospital defined as a competing event in the time until discharge analyses. Sensitivity analysis was conducted on associations between data on time to discharge alive and length of mechanical ventilation, and energy and protein deficit to include only those patients who stayed in ICU for a full eight days: this analysis was undertaken in order to account for those patients who had a short stay in ICU and therefore a better outcome, but for whom nutrition support might not be indicated. Statistical significance was considered as a *p* value <0.05.

## Results

### Demographics

From 17,689 patients from 592 ICUs for whom data were available, data were extracted for all patients with a primary diagnosis of head trauma (with and without other traumatic injuries). Diagnosis of head trauma was recorded in 1,045 patients, who were included for analysis. These patients were admitted to one of 341 ICUs from 31 countries with each ICU contributing an average of 3.1 (2.4) patients. The majority of patients were admitted to ICUs in the USA (30 %), Australia (14 %), and Canada (12 %). Data were collected from most patients for the entire 12 study days (60 %), with a total of 10,558 study days recorded. Patient demographics are shown in Table 1.

**Table 1:** Patient demographics

	Total	Patient sample
Age (y), mean (SD)	44.5 (19.7)	1,045
Sex (male) n, (%)	815 (78)	1,045
Initial GCS:		251
GCS 13–15, n (%)	18 (7)	
GCS 10–12, n (%)	23 (9)	
GCS 6–9, n (%)	96 (38)	
GCS <6, n (%)	114 (45)	
APACHE II, mean (SD)	19.5 (6.9)	1,038
SOFA score, mean (SD)	7.6 (3.1)	257
Weight (kg), mean (SD)	77.4 (17.3)	1,045
Height (m), mean (SD)	1.73 (0.09)	1,040
Body mass index (kg/m^2^), mean (SD)	25.7 (5.2)	1,040
Underweight (<18.5 kg/m^2^), n (%)	30 (3)	
Healthy (18.5–24.9 kg/m^2^), n (%)	519 (50)	
Overweight (25–29.9 kg/m^2^), n (%)	348 (34)	
Obese (>30 kg/m^2^), n (%)	143 (14)	

GCS and SOFA data were collected in 2013 only. *GCS* Glasgow Coma Scale, *APACHE II* Acute Physiology and Chronic Health Evaluation II, *SOFA* Sequential Organ Failure Assessment

### Feeding protocols

Most patients (863/1045; 83 %) were from an ICU where a bedside feeding protocol was used to allow the nurse to advance or withhold enteral feeds. Protocols contained algorithms for: motility agents (n = 667, 64 %); small bowel feeding (n = 506, 48 %); withholding nutrition for procedures (n = 480, 46 %); head of bed elevation (n = 698, 67 %); and gastric residual volume (GRV) thresholds (n = 823, 79 %). In those ICUs with a GRV algorithm, the median GRV threshold was 250 (range 50–500) ml and the mode threshold was 200 ml (n = 331, 40 %).

### Nutritional assessment and prescription

The majority of patients (n = 871, 83 %) were admitted to an ICU that employed a dietician, of which 40 % had at least one full-time dietician. During the years that data relating to baseline nutrition assessments were recorded (2010–2013), 85 % (n = 443/519) of patients had a baseline nutrition assessment completed. This assessment included documentation of body weight in half of patients (n = 260) and of height in 46 % of patients (n = 237).

A variety of methods were used to estimate energy requirements. The most frequently utilised method was a weight-based approach, for example 25 kcal/kg, which was used in 49 % of patients (n = 508). Equations were used in 432 patients (42 %), the most popular of these being Harris Benedict and Schofield. Eleven patients (1 %) had their energy expenditure estimated through indirect calorimetry. The mean amount of energy and protein prescribed daily was 1,958 (376) kilocalories and 98.7 (26.6) grams respectively, equivalent to 25.9 (4.9) kcal/kg/day and 1.29 (0.3) g/kg/day.

### Nutritional delivery

At some point during the study period the majority of patients (94 %, n = 983) received enteral nutrition (EN), 13 % (n = 138) received parenteral nutrition (PN), and 20 % (n = 207) ingested nutrient orally. Sixteen patients (2 %) received no nutrition during the study period. Twenty-four percent (n = 239) of patients had EN commenced on day 1 of ICU admission, 41 % (n = 404) on day 2, and 20 % (n = 195) on day 3. The mean time from ICU admission to initiation of EN was 35.5 (32.7) hours.

Patients often received more than one concentration of EN formula; however, a 1 kcal/ml formula was the most common and was delivered for 53 % of all EN prescriptions. Twenty-five percent of prescriptions were of a concentrated enteral formula that provided 1.5 kcal/ml or greater. One-hundred and twenty-two (12 %) patients received glutamine during their ICU admission with a mean daily dose of 22 (11) grams. Glutamine was usually delivered via the enteral route (66 % of patients).

Of those patients receiving EN the location of the feeding tube was reported for 926 (94 %) patients. Gastric feeding was the most common route of EN, and used exclusively in 67 % (n = 620) of patients; 11 % (n = 101) of patients were exclusively fed via post-pyloric tubes, and 22 % (n = 205) received EN through a combination of gastric and post-pyloric routes. Gastrokinetic drugs were frequently prescribed; 70 % of patients (n = 713) received a gastrokinetic drug at some stage. The prevalence of gastrokinetic drug use varied according to day of admission, with 29 % (n = 185) of patients receiving gastrokinetics on day 1, 50 % (n = 415) on day 2, and a peak of 61 % (n = 556) by day 5. Even at nutritional data censor (i.e., day 12) 56 % (n = 320) of patients were receiving gastrokinetics. Metoclopramide was the most commonly prescribed gastrokinetic drug and was administered to 38 % (n = 400) of patients.

### Interruptions

**Fig. 1:**
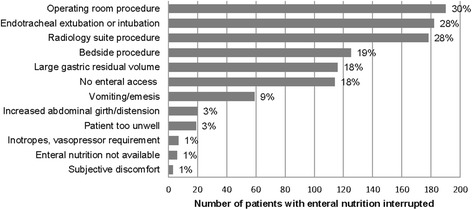
Reasons for interruptions to enteral nutrition support (n = 644)

Of the patients who received EN, 66 % (n = 644) had interruptions to feeds at least once during the study period. Thirty percent (n = 191) had interruptions to feeding on just one day, 21 % (n = 133) had interruptions on two days, 16 % (n = 103) had interruptions on three days, and 34 % (n = 217) had four or more days where feeding was interrupted. There were various reasons for interruptions to enteral feeds (Fig. 1). From 2009 to 2013, the number of hours of interruptions to EN were collected, with a mean duration of 25.3 (range 0.2–120) hours per patient, equivalent to 2.6 (range 0.1–18.8) hours per day.

### Energy and protein intake and deficit

**Fig. 2:**
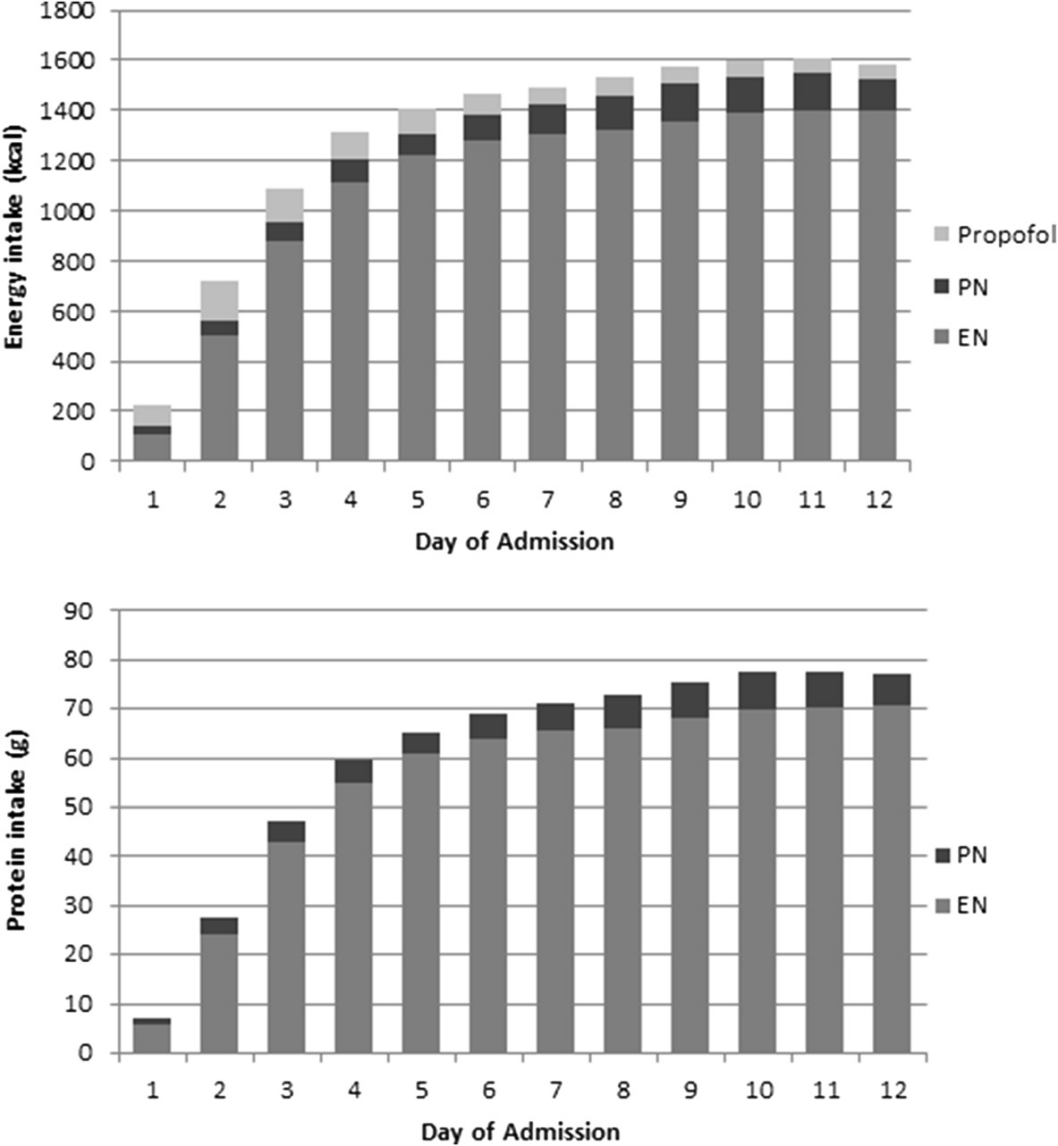
Mean daily energy and protein contribution from enteral and parenteral nutrition and propofol. *PN* parental nutrition, *EN* enteral nutrition

**Fig. 3:**
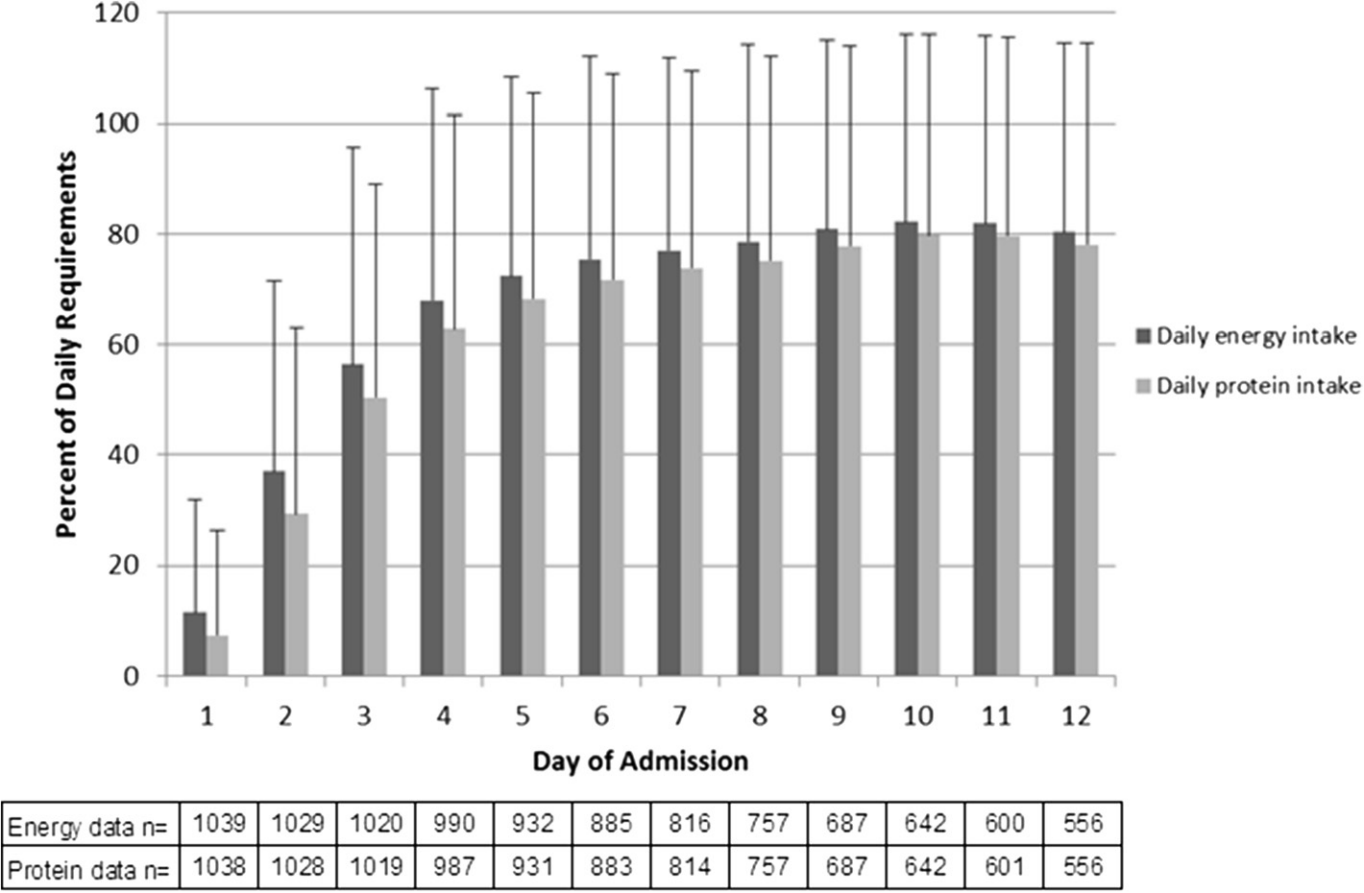
Mean daily energy and protein intake as a percent of requirements per study day

Energy and protein were received from various sources (Fig. 2). Over half of the patients received propofol (59 %, n = 618), which provided a mean of 161 (165) kilocalories of additional energy per day. The mean amount of energy received from EN was 974 (524) kcal/day, and 86 (269) kcal/day from PN. The mean delivery of energy and protein to patients from all sources was 1154 (525) kcal/day and 52 (26) g/day, respectively; equivalent to 15.3 (7.2) kcal/kg/day and 0.69 (0.4) g/kg/day. The daily mean energy and protein deficit was 803 (527) kilocalories and 46 (30) grams, respectively. Nutrition from all sources met an average of 58 (range 0–166) % of estimated energy requirements and 53 (range 0–390) % of protein requirements. Daily intake data is shown in Fig. 3.

### Glucose control

Eighty-nine percent (n = 926) of patients were from an ICU that contained a protocol to monitor blood glucose and administer insulin. In those protocols that contained a blood glucose target, the median lower blood glucose target was 4.5 (range 3.0–8.3) mmol/l and the upper blood glucose target was 8.3 (range 5.3–15.0) mmol/l. For 700 patients from 2009–2013 the mean highest blood glucose recorded in the first 24 hours of ICU admission was 9.8 (3.3) mmol/l and the lowest was 6.5 (1.9) mmol/l. The mean morning blood glucose during the study period was 7.5 (1.3) mmol/l. An episode of hypoglycaemia occurred in 9 % (n = 90) of patients. Insulin was provided in 59 % of cases (n = 611), of which the average daily insulin dose provided was 36.5 (36.2) units.

### Outcomes

Of the 1,045 patients, 135 (13 %) died in ICU, 38 (4 %) died after ICU discharge in hospital, and 872 (83 %) survived to hospital discharge or were alive in hospital at day 60. Male patients were more likely to survive a head trauma than females (odds ratio (OR) 0.66; 95 % CI 0.46, 0.96; p = 0.026). The median ICU length of stay in survivors was 13.1 (IQR 7.9–21.6) days, and the median hospital length of stay was 29.7 (IQR 17.9–57.1) days. The median length of time during which patients required mechanical ventilation was 9.2 (IQR 4.8–15.4) days.

Patients from an ICU that utilised a feeding protocol had greater energy and protein intakes per body weight than those without (*p* <0.001, 0.002 respectively) and were more likely to survive (OR 0.65; 95 % CI 0.42, 0.99; *p* = 0.043; Table 3). When the feeding protocol contained guidance on motility agents and small bowel feeding there was a smaller energy and protein deficit (Table 2). Patients from an ICU with a feeding protocol that contained details on GRVs had smaller protein deficit, but no difference in energy delivery.

**Table 2:** Relationship between ICU and patient nutritional variables and energy and protein deficit

		Energy deficit (kcal/d)			Protein deficit (g/d)		
		Mean	Standard deviation	*p*	Mean	Standard deviation	*p*
Bedside feeding protocol	Yes	788	528	0.223	45.2	30.2	0.129
	No	877	518		52.0	30.0	
Bedside feeding protocol includes gastric residual volumes	Yes	783	532	0.094	44.7	30.0	**0.038**
	No	878	505		52.6	30.4	
Bedside feeding protocol includes motility agents	Yes	732	507	**<.001**	42.1	29.3	**<.001**
	No	930	539		54.1	30.5	
Bedside feeding protocol includes small bowel feeding	Yes	720	507	**<.001**	41.0	29.0	**0.001**
	No	883	535		51.6	30.5	
Bedside feeding protocol includes withholding for procedures	Yes	755	543	0.076	42.1	29.9	**0.008**
	No	844	511		50.1	30.1	
Bedside feeding protocol includes head of bed elevation	Yes	791	529	0.547	46.1	31.5	0.969
	No	827	524		47.1	27.6	
Dietician working in the ICU	No dietician	658	524	0.027*	29.9	26.1	**<.001**^
	<1 FTE	844	539		48.6	29.5	
	≥1 FTE	820	505		51.3	30.6	
Timing of initiation of EN	By day 1	512	431	**<.001** ^**#**^	31.3	23.6	**<.001** ^**#**^
	Day 2–4	811	465		47.8	29.0	
	After day 4	1167	551		62.5	31.3	
Nutritional support route	EN only	734	468	**0.023** ^**+**^	44.4	28.2	**<.001** ^**+**^
	PN only	624	215		26.7	25.6	
	EN + PN	602	524		31.6	30.3	
Formula density (patients with >75 % of days at one density)	≤1 kcal/ml	728	453	**0.011** ^**π**^	41.9	27.7	**0.006** ^**π**^
	>1– < 2 kcal/ml	816	540		48.7	30.6	
	≥2 kcal/ml	586	491		47.0	34.9	

*None significantly different from <1 full-time equivalent dietician (*FTE*). ^None significantly different from both <1 FTE and ≥1 FTE. ^#^All groups significantly different to all others. ^+^Enteral nutrition + parental nutrition (*EN + PN*) significantly different from EN only. ^π^ ≤ 1 kcal/ml’ significantly different from > 1– < 2 kcal/ml. Significant p-values denoted in bold text

Earlier initiation of EN was significantly associated with a reduction in energy and protein deficit (*r* = 0.32 and 0.27 respectively, *p* <0.001). While the point estimate indicated reduced mortality when EN was commenced on day 1 when compared to days 2–4 or day 5 or later, this was not significant (Table 3). Greater duration of EN interruptions increased both energy and protein deficit (*r* = 0.219 and 0.218 respectively, *p* <0.001). Energy and protein deficits were reduced when EN and PN were used in combination, compared with EN alone (*p* = 0.023 and <0.001 respectively, Table 3).

**Table 3:** Relationship between unadjusted ICU and patient nutritional variables and mortality

		Survivors		Non-survivors		Odds ratio	95 % CI	*p*
		n	%	n	%			
Sex	Male	691	84.8	124	15.2	0.66	0.46, 0.96	**0.029**
	Female	181	78.7	49	21.3	ref	-	
EN interrupted	Yes	548	85.1	96	14.9	0.89	0.62, 1.27	0.506
	No	283	83.5	56	16.5	ref	-	
Bedside feeding protocol	Yes	730	84.6	133	15.4	0.65	0.42, 0.99	**0.043**
	No	142	78.0	40	22.0	ref	-	
Bedside feeding protocol includes gastric residual volumes	Yes	699	84.9	124	15.1	0.63	0.43, 0.92	**0.016**
	No	173	77.9	49	22.1	ref	-	
Bedside feeding protocol includes motility agents	Yes	562	84.3	105	15.7	0.85	0.60, 1.20	0.360
	No	442	82.0	97	18.0	ref	-	
Bedside feeding protocol includes small bowel feeding	Yes	430	85.0	76	15.0	0.81	0.58, 1.12	0.201
	No	442	82.0	97	18.0	ref	-	
Bedside feeding protocol includes withholding for procedures	Yes	399	83.1	81	16.9	1.04	0.75, 1.46	0.800
	No	473	83.7	92	16.3	ref	-	
Bedside feeding protocol includes head of bed elevation	Yes	586	84.0	112	16.0	0.90	0.63, 1.27	0.540
	No	286	82.4	61	17.6	ref	-	
Dietician working in the ICU	No dietician	139	79.9	35	20.1	ref	-	**-**
	<1 FTE	427	86.1	69	13.9	0.64	0.41, 0.99	**0.047**
	≥1 FTE	302	81.4	69	18.6	0.91	0.58, 1.41	0.667
Formula density (patients with >75 % of days at one density)	≤1 kcal/ml	391	85.2	68	14.8	ref	-	-
	>1–<2 kcal/ml	275	83.6	54	16.4	1.13	0.77, 1.67	0.541
	≥2 kcal/ml	16	69.6	7	30.4	2.52	0.998, 6.34	0.051
Timing of initiation of EN	Day 1 or prior to ICU admission	210	87.9	29	12.1	ref	-	-
	Day 2–4	569	83.7	111	16.3	1.41	0.91, 2.19	0.122
	Day 5 or later	52	81.3	12	18.8	1.67	0.80, 2.50	0.173

*FTE* full-time equivalent, *EN* enteral nutrition

There was a non-significant association between at least one recorded episode of hypoglycaemia and higher risk of mortality (OR 1.6; 95 % CI 0.96, 2.7; *p* = 0.073). Any hypoglycaemic event was associated with a reduced probability of being discharged alive from ICU: (hazard ratio (HR) 0.78; 95 % CI 0.60, 0.99; *p* = 0.043) and hospital: (HR 0.78; 95 % CI: 0.58, 1.03; *p* = 0.082).

A greater energy and protein deficit (OR per 100 kcal/day) was associated with longer times until discharge alive from ICU (energy: *p* <0.001, protein: *p* = 0.001) and hospital (energy: *p* = 0.002, protein: *p* = 0.024) (Table 4). A greater energy and protein deficit was also associated with longer time receiving mechanical ventilation (OR per 100 kcal/day, *p* <0.001; Table 4). However, when a sensitivity analysis was performed to include only those patients who stayed in ICU for a full eight days a statistically significant relationship only remained for energy deficit on time to discharge alive from hospital and length of mechanical ventilation (*p* = 0.001 n = 816, and *p* = 0.004 n = 732 respectively; Table 4). In an unadjusted analysis there was a significant protective effect of energy (per 10 kcal/kg/day), but not protein, delivery on mortality (energy: OR 0.79; 95 % CI 0.63, 0.998; *p* = 0.048). However, in the adjusted analysis neither energy nor protein delivery affected mortality (energy: OR 0.76; 95 % CI 0.48, 1.22; *p* = 0.256, protein: OR 1.01; 95 % CI 0.92, 1.118; *p* = 0.868).

**Table 4:** Relationship between energy and protein deficit and length of mechanical ventilation and time to discharge alive

Variable		Time until discharged alive from ICU (days)		Time until discharged alive from hospital (days)		Length of mechanical ventilation (days)	
		Hazard ratio (95 % CI)	*p*	Hazard ratio (95 % CI)	*p*	Hazard ratio (95 % CI)	P
All patients		n = 1027 energy, 1026 protein		n = 1027 energy, 1026 protein		n = 896	
	Energy deficit (kcal/day)	1.04 (1.02, 1.05)	<0.001	1.03 (1.01, 1.04)	0.002	1.07 (1.05, 1.08)	<0.001
	OR is per 100 kcal/day						
	Protein deficit (g/day)	1.02 (1.01, 1.04)	0.001	1.02 (1.002, 1.03)	0.024	1.05 (1.03, 1.06)	<0.001
	OR is per 5 g/day						
Patients who stayed at least 8 days		n = 816		n = 816		n = 732	
	Energy deficit (kcal/day)	1.01 (0.99, 1.02)	0.948	1.02 (1.01, 1.04)	0.001	1.02 (1.01, 1.04)	0.004
	OR is per 100 kcal/day						
	Protein deficit (g/day)	0.99 (0.98, 1.01)	0.275	1.01 (0.99, 1.03)	0.316	1.01 (0.996, 1.03)	0.161
	OR is per 5 g/day						

Adjusted for age, sex, region, Acute Physiology and Chronic Health Evaluation II score, body mass index category, admission category, and clustering of patients within ICUs. *OR* odds ratio

## Discussion

The purpose of our study was to describe international nutrition support practices and factors that influence nutrient delivery, and evaluate relationships between nutrient delivery and clinical outcomes in critically ill head-injured patients. Our dataset of >10,000 patient days from 1,045 patients provided a unique opportunity to evaluate these variables.

The most significant finding was the observation that head-injured patients were significantly underfed, receiving just 58 % of their estimated energy and 53 % of their estimated protein requirements. These data are consistent with studies in cohorts of mixed medical-surgical ICU patients [17–19], but are lower than in other cohorts of patients that are considered to be hypermetabolic; trauma, neurosurgical and burns patients have been reported to meet between 67 and 76 % of their nutritional requirements [20–22]. This observation is of interest, given that gastrointestinal dysmotility and delayed gastric emptying occurs frequently in all these conditions [23]. It may be that in other hypermetabolic conditions, such as burns, the provision of nutritional support is of greater priority and is a focus of treatment. Additionally, many of the barriers associated with feeding after head injury, such as inadvertent removal of feeding tubes, may be more prevalent after the time when the patient is no longer sedated, and hence adequacy of nutrition over the longer term, after ICU discharge, may be more important [24]. Additionally, ICU admission and discharge days were counted as complete days and therefore achievement of 100 % of nutritional requirements on these days is unlikely to be desirable.

Greater energy and protein deficits were significantly associated with longer times to discharge alive from ICU and hospital. However, when we undertook sensitivity analysis to include only those patients with an ICU stay ≥8 days, only energy deficit was associated with a delayed time to discharge alive from hospital and length of mechanical ventilation. A recent meta-analysis on the delivery of enteral nutrition to critically ill patients reported no significant interaction between energy and protein intake on ICU, or hospital length of stay [25]. However, interpretation of the latter study is limited as group data do not enable investigators to analyse death and length of stay as dependent variables. A strength of our study is that appropriate adjustment was made for competing variables (death in ICU and ICU length of stay) [26]. We hypothesized that nutritional therapy could be of particular benefit to patients with head injury as the injury itself generally results in longer length of stay when compared to critically ill counterparts, which means that energy and protein intake may have a greater capacity to influence clinical outcomes. Correspondingly, research specific to head injury has shown that nutritional interventions, such as early when compared to delayed nutrition support, can reduce hospital and ICU length of stay [27–29]. Greater energy deficit, even after sensitivity analysis, was also associated with a longer time requiring mechanical ventilation. Whether these relationships are true, or are a result of underlying unadjusted factors such as severity of injury, requires further investigation.

When adjusted for evaluable nutrition days and clinical characteristics we did not observe relationships between energy and protein intakes and mortality. Our findings are contradictory to analyses by Hartl and colleagues who reported in 797 severely head-injured patients improved survival between seven and 14 days of admission with each 10 kcal/kg body weight/day increase in the maximum amount of energy received in the first 5 to 7 days of ICU admission [11]. We believe variations between inclusion criteria and statistical analyses may account for the different results from our study. Data on severity of head injury, which may increase energy expenditure and be independently associated with outcome [30], were not collected in earlier INSs and hence not adjusted for in our analysis, whereas Hartl and colleagues adjusted for potentially important factors, such as hypotension, pupil status, and findings on computed tomography, which were not collected as part of the INS. However, Hartl and colleagues did not account for other confounders, such as evaluable nutrition days or BMI, the study was conducted in a single region, and only reported deaths between day seven and day 14, and the latter factor has the capacity to bias the results. The reasons for these contradictory results require further exploration. Nonetheless, because our analyses are based on those deemed to be the most appropriate at present [16], we believe it adds incrementally to the body of evidence on nutrition support for head-injured patients.

We recognize however that optimal energy and protein targets after head injury are unknown. The extent of hypermetabolism and catabolism are dependent on ventilation status, sedation, severity of head injury, and posturing, which makes it challenging to accurately estimate energy expenditure and protein needs for an individual patient [13]. Particularly after head injury, generalised predictive equations, as were used for the majority of patients in this dataset, have been shown to be somewhat inaccurate in determining nutritional needs when compared to more direct yet invasive methodology such as indirect calorimetry and nitrogen balance studies [31]. Additionally, these predictive equations and a weight-based approach incorporate patient’s body weight, yet obtaining a weight can be challenging and inaccurate in the intensive care setting; a weight was only documented in half of those patients with a nutritional assessment. While these equations are imprecise, and there are no definitive recommendations for energy and protein requirements, energy and protein delivery to meet predicted needs of 100–140 % of resting energy expenditure and 2.0–2.5 g protein/kg/day are suggested [13, 32]. The energy and protein intakes we observed, 15.3 (7.2) kcal/kg/day and 0.69 (0.4) g/kg/day, are substantially lower than these suggestions. Furthermore, because only 2 % of patients received >1.5 g protein/kg/day, and no patient received more than 2 g/kg/day, we cannot exclude the possibility that a greater protein intake is associated with benefit (or harm) to attenuate the catabolic response and hence influence survival. An analysis from the same international dataset including all medical-surgical ICU patients reported that achieving ≥80 % of prescribed protein intake was associated with reduced mortality [33]. Hence, further research with higher energy and protein intakes are required. Similarly, the interplay between energy and protein intakes could be important but we were not able to adequately assess these interactions with this dataset.

We did observe substantial energy and protein deficits, even early after head injury. A number of clinical barriers hinder adequate feeding, so strategies that assist to improve nutritional deficits in this population may be of importance. As reported previously and reiterated in this analysis, ICUs with a feeding protocol in place are able to significantly improve energy and protein delivery and should be commonplace [34, 35]. We observed that patients from an ICU where the feeding protocol contained the use of gastric residual volumes had lower protein intakes. It is plausible that these ICUs also have differences in other practices, such as greater use of concentrated formulas, or higher propofol intakes that may have accounted for this relative lower protein intake in comparison to caloric intake. It is also plausible that this is a spurious finding that requires investigation through well-designed randomized trials. In our study, patients from ICUs where the feeding protocol contained guidance on motility agents had higher energy intakes. It is well-documented that gastric dysmotility occurs frequently after head injury [5, 36]; 70 % of patients in this study received gastrokinetic agents, suggesting that the majority of patients experienced enteral feed intolerance or clinicians were sufficiently concerned to prescribe these drugs. In addition to the use of gastrokinetic drugs another strategy that has been shown to increase nutrition delivery is the use of a concentrated enteral formula [37, 38]. In our cohort, only 20 % of patients received a 1.5 kcal/ml enteral formula, and only 5 % received a 2 kcal/ml formula at any time point. While energy-dense feeds ≥2 kcal/ml may have the capacity to slow gastric emptying and worsen feed intolerance [39], the utilization of a concentrated enteral formula to improve energy intakes after head injury should be investigated. However, we also observed that use of energy-dense formulas was associated with a greater protein deficit, likely due to the addition of fat to these formulas rather than protein to increase the calorie content, and this requires consideration by clinicians when prescribing particular feeds. Additionally, protocols, such as the PEP uP protocol (efficacy of enhanced Protein-Energy Provision via the enteral roUte in critically ill Patients), that have been shown to improve energy and protein delivery in a critically ill population, could be utilized [34].

Current guidelines recommend early initiation of nutritional therapy, with achievement of goal requirements by day seven [13]. However, in our study nutritional intake remained suboptimal by day seven, and longer time to initiation of feeds was associated with greater energy and protein deficits. Additionally, multiple interruptions to enteral nutrition occurred, which reduced intake [17]. Nutrition delivery was primarily interrupted due to fasting for procedures and intubation/extubation of the trachea. While these interruptions may be largely unavoidable, exploration and minimization of fasting times could be considered [21]. Additionally, the presence of a dietician had conflicting effects on energy and protein delivery, with the time spent in the ICU influencing nutrient intake. These results need to be cautiously interpreted, as there are several confounding variables that were not measured. Previous studies have suggested that a full-time dietician is required to improve energy delivery [40]. Men were more likely to survive than women. This outcome has been reported in other traumatic conditions, and the mechanism/s behind this result requires further exploration [22].

A strength of our study is that it is the largest prospective observational study to evaluate nutrient delivery in critically ill head-injured patients from an international perspective. A multi-centre study enables greater generalizability of data, and having larger numbers of patients minimizes the effect of between-patient variation. Previous studies have been conducted retrospectively, in single centres, or have included small numbers of patients. However, in our study patients were significantly underfed compared to prescribed requirements, and few patients met their estimated nutritional needs over the 12-day period, so it may be difficult to fully assess the influence of adequate energy and protein intakes on outcomes. This is particularly true for protein, as in our study the greatest intake was 1.83 g/kg/day, which is below the current recommendations of 2.0–2.5 g/kg/day [13, 32]. Another limitation of our study is that we did not have access to data describing the severity of the head injury and presence of other injuries. These parameters have the capacity to alter metabolic demands and may influence outcomes. Unfortunately, these details were not collected in this prospective survey. In addition, the sample size we studied may have been insufficient to detect any mortality difference. Given that most ICUs contributed just three patients to this dataset, recommendations for individual site-level processes cannot be deduced. Additionally, all ICUs participated in the INS on a voluntary basis which may have attracted those ICUs with an interest in nutrition and hence influence the generalizability of the results. Lastly, nutrition may be able to influence other important outcomes, such as repeat hospitalization, functional status, and quality of life, which were not explored in this dataset. Therefore, future studies should consider the influence of nutrition over the longer-term on morbidity outcomes in addition to mortality to enable a greater understanding of the role nutrition plays in recovery from a head injury.

## Conclusions

We observed that delivery of energy and protein to critically ill head-injured patients is considerably less than recommended. Greater energy and protein deficits were associated with delays to discharge alive from ICU and hospital. However, we did not observe a relationship between these deficits and increased mortality. Further research into the optimal dose of energy and protein to enhance the long-term recovery of patients after head injury is warranted. In the meantime, our study suggests that efforts to increase nutritional intake and prevent energy and protein debt in these patients appear justified.

## Key messages

- This is the largest international study on energy and protein delivery in critically ill head-injured patients
- Patients were significantly underfed receiving just 58 % estimated energy and 53 % protein requirements
- Greater energy and protein deficits were associated with a delay to discharge alive from ICU and hospital
- Efforts to increase intake to prevent energy and protein debt such as feeding protocols and minimisation of interruptions should be considered
- Future research should explore the effect of adequate energy and protein intakes, including longer-term delivery, on morbidity outcomes in addition to mortality

## Abbreviations

APACHE II: Acute Physiology and Chronic Health Evaluation II
BMI: body mass index
EN: enteral nutrition
GCS: Glasgow Coma Scale
GRV: gastric residual volume
HR: hazards ratio
ICU: Intensive Care Unit
INS: International Nutrition Survey
IQR: interquartile range
OR: odds ratio
PN: parenteral nutrition
SD: standard deviation
SOFA: sequential organ failure assessment

## References

[1] Foley N, Marshall S, Pikul J, Salter K, Teasell R (2008). Hypermetabolism following moderate to severe traumatic acute brain injury: a systematic review. J Neurotrauma.

[2] Genton L, Pichard C (2011). Protein catabolism and requirements in severe illness. Int J Vitam Nutr Res.

[3] Cartwright MM (2004). The metabolic response to stress: A case of complex nutrition support management. Crit Care Nurs Clin North Am.

[4] Dickerson RN, Pitts SL, Maish GO, Schroeppel TJ, Magnotti LJ, Croce MA (2012). A reappraisal of nitrogen requirements for patients with critical illness and trauma. J Trauma Acute Care Surg.

[5] Tan M, Zhu J-C, Yin H-H (2011). Enteral nutrition in patients with severe traumatic brain injury: reasons for intolerance and medical management. Br J Neurosurg.

[6] Alhashemi HH (2010). Dysphagia in severe traumatic brain injury. Neurosci.

[7] Krakau K, Hansson A, Karlsson T, de Boussard CN, Tengvar C, Borg J (2007). Nutritional treatment of patients with severe traumatic brain injury during the first six months after injury. Nutr.

[8] Lim SL, Ong KC, Chan YH, Loke WC, Ferguson M, Daniels L (2012). Malnutrition and its impact on cost of hospitalization, length of stay, readmission and 3-year mortality. Clin Nutr.

[9] Wang X, Dong Y, Han X, Qi XQ, Huang CG, Hou LJ (2013). Nutritional support for patients sustaining traumatic brain injury: a systematic review and meta-analysis of prospective studies. PLoS One.

[10] Costello LS, Lithander FE, Gruen RL, Williams LT (2014). Nutrition therapy in the optimisation of health outcomes in adult patients with moderate to severe traumatic brain injury: findings from a scoping review. Injury.

[11] Hartl R, Gerber LM, Ni Q, Ghajar J (2008). Effect of early nutrition on deaths due to severe traumatic brain injury. J Neurosurg.

[12] Heyland DK, Dhaliwal JW, Gramlich L, Dodek P (2003). Canadian clinical practice guidelines for nutrition support in mechanically ventilated, critically ill adult patients. JPEN.

[13] Bratton S, Chestnut R, Ghajar J, McConnell Hammond F, Harris O, Hartl R, et al. Guidelines for the management of severe traumatic brain injury. XII. Nutrition. J Neurotrauma. 2007;24 Suppl 1:S77–82. 10.1089/neu.2006.9984 17511551

[14] Jacobs D, Jacobs D, Kudsk K, Moore F, Oswanski M, Poole G (2004). Practice management guidelines for nutritional support of the trauma patient. J Trauma Inj Infect Crit Care..

[15] Alberda C, Gramlich L, Jones N, Jeejeebhoy K, Day A, Dhaliwal R (2009). The relationship between nutritional intake and clinical outcomes in critically ill patients: results of an international multi-center observational study. Intensive Care Med..

[16] Heyland DK, Cahill N, Day AG (2011). Optimal amount of calories for critically ill patients: depends on how you slice the cake!. Crit Care Med..

[17] De Beaux I, Chapman M, Fraser R, Finnis M, De Keulenaer B, Liberalli D (2001). Enteral nutrition in the critically ill: A prospective survey in an Australian intensive care unit. Anaesth Intensive Care..

[18] De Jonghe B, Appere-De-Vechi C, Fournier M, Tran B, Merrer J, Melchior JC (2001). A prospective survey of nutritional support practices in intensive care unit patients: What is prescribed? What is delivered?. Crit Care Med..

[19] Reid C (2006). Frequency of under- and overfeeding in mechanically ventilated ICU patients: causes and possible consequences. J Human Nutr Diet..

[20] Kim H, Shin JA, Shin JY, Cho OM (2010). Adequacy of nutritional support and reasons for underfeeding in neurosurgical intensive care unit patients. Asian Nurs Res (Korean Soc Nurs Sci).

[21] Morgan LM, Dickerson RN, Alexander KH, Brown RO, Minard G (2004). Factors causing interrupted delivery of enteral nutrition in trauma intensive care unit patients. Nutr Clin Pract.

[22] Czapran A, Headdon W, Deane AM, Lange K, Chapman MJ, Heyland DK (2015). International observational study of nutritional support in mechanically ventilated patients following burn injury. Burns.

[23] Chapman MJ, Nguyen NQ, Deane AM (2011). Gastrointestinal dysmotility: clinical consequences and management of the critically ill patient. Gastroenterol Clin North Am.

[24] Brooke MM, Barbour PG, Cording LG, Tolan C, Bhoomkar A, McCall GW (1989). Nutritional status during rehabilitation after head injury. J Neuro Rehab.

[25] Tian F, Wang X, Gao X, Wan X, Wu C, Zhang L (2015). Effect of initial calorie intake via enteral nutrition in critical illness: a meta-analysis of randomised controlled trials. Crit Care..

[26] Wolkewitz M, Beyersmann J, Gastmeier P, Schumacher M (2009). Modeling the effect of time-dependent exposure on intensive care unit mortality. Intensive Care Med.

[27] Chourdakis M, Kraus MM, Tzellos T, Sardeli C, Peftoulidou M, Vassilakos D (2012). Effect of early compared with delayed enteral nutrition on endocrine function in patients with traumatic brain injury: An open-labeled randomized trial. JPEN.

[28] Vitaz TW, Jenks J, Raque GH, Shields CB (2003). Outcome following moderate traumatic brain injury. Surg Neurol.

[29] Chiang Y-H, Chao D-P, Chu S-F, Lin H-W, Huang S-Y, Yeh Y-S (2012). Early enteral nutrition and clinical outcomes of severe traumatic brain injury patients in acute stage: A multi-center cohort study. J Neurotrauma.

[30] Robertson CS, Clifton GL, Grossman RG (1984). Oxygen utilization and cardiovascular function in head-injured patients. Neurosurgery.

[31] Sunderland PM, Heilbrun MP (1992). Estimating energy expenditure in traumatic brain injury: comparison of indirect calorimetry with predictive formulas. Neurosurgery.

[32] Matters K, Murray EJ, Mok V, Flower O (2014). Protein requirements in traumatic brain injury: a systematic review. Australas J Neurosci.

[33] Nicolo M, Heyland DK, Chittams J, Sammarco T, Compher C (2015). Clinical outcomes related to protein delivery in a critically ill population: a multicenter, multinational observation study. JPEN.

[34] Heyland DK, Cahill NE, Dhaliwal R, Wang M, Day AG, Alenzi A (2010). Enhanced protein-energy provision via the enteral route in critically ill patients: a single center feasibility trial of the PEP uP protocol. Crit Care..

[35] Adam S, Batson S (1997). A study of problems associated with the delivery of enteral feed in critically ill patients in five ICUs in the UK. Intensive Care Med..

[36] Krakau K, Omne-Pontén M, Karlsson T, Borg J (2006). Metabolism and nutrition in patients with moderate and severe traumatic brain injury: A systematic review. Brain Inj.

[37] Sheean PM, Peterson SJ, Zhao W, Gurka DP, Braunschweig CA (2012). Intensive medical nutrition therapy: methods to improve nutrition provision in the critical care setting. J Acad Nutr Diet.

[38] Peake SL, Davies AR, Deane AM, Lange K, Moran JL, O'Connor SN (2014). Use of a concentrated enteral nutrition solution to increase calorie delivery to critically ill patients: a randomized, double-blind, clinical trial. Am J Clin Nutr.

[39] Kar P, Plummer MP, Chapman MJ, Cousins CE, Lange K, Horowitz M, et al*.* Energy-dense formulae may slow gastric emptying in the critically ill. JPEN. 2015. E pub ahead of print. doi:10.1177/0148607115588333. 10.1177/0148607115588333 26038421

[40] Soguel L, Revelly JP, Schaller MD, Longchamp C, Berger MM (2012). Energy deficit and length of hospital stay can be reduced by a two-step quality improvement of nutrition therapy: the intensive care unit dietitian can make the difference. Crit Care Med.

